# Antibacterial and anti-biofilm activity of plumbagin against multi-drug resistant clinical bacterial isolates

**DOI:** 10.15537/smj.2022.43.11.20220446

**Published:** 2022-11

**Authors:** Mohammad A. Alfhili, Irfan Ahmad, Yasser Alraey, Abdulaziz Alangari, Taha Alqahtani, Ayed A. Dera

**Affiliations:** *From the Chair of Medical and Molecular Genetics Research (Alfhili), Department of Clinical Laboratory Sciences, from the Department of Clinical Laboratory Sciences (Alangari), College of Applied Medical Sciences, King Saud University, Riyadh; from the Department of Clinical Laboratory Sciences (Ahmad, Alraey, Dera), College of Applied Medical Sciences, and from the Department of Pharmacology (Alqahtani), College of Pharmacy, King Khalid University, Abha, Kingdom of Saudi Arabia.*

**Keywords:** Plumbagin, *P. zeylanica*, antibacterial, biofilm, *S. aureus*, *E. coli*

## Abstract

**Objectives:**

To evaluate the antibacterial activity of plumbagin (PGN) against multidrug resistance (MDR) clinical isolates.

**Methods:**

This study was carried out at the Department of Clinical Lab Sciences, King Khalid University from October 6, 2021 to December 14, 2021. We investigated the antibacterial and anti-virulence activity of PGN against MDR Gram-negative (*Escherichia coli*, *Klebsiella pneumoniae*, *Salmonella Typhi*, and *Pseudomonas aeruginosa*) and Gram-positive (*Staphylococcus aureus [S. aureus]*, *Staphylococcus saprophyticus [S. saprophyticus]*, *Streptococcus pyogenes*, and *Enterococcus faecalis*) clinical bacterial isolates. Agar well diffusion, microdilution assay, colony count method, biofilm formation, and time-kill kinetics were employed to probe the MIC, MBC, and anti-virulence activity of PGN.

**Results:**

Plumbagin inhibited the growth of all tested isolates, with *S. saprophyticus* exhibiting the highest sensitivity. MIC values ranged from 0.029 to 0.117 µg/mL whereas MBC ranged from 0.235 to 0.94 µg/mL, with 79% to 99% growth inhibition. Moreover, all tested isolates showed a marked decrease in biofilm formation, with *S. saprophyticus* and *S. aureus* being the most sensitive.

**Conclusion:**

Plumbagin is a stand-alone, broad spectrum antibacterial with promising potential against the rising threat of antimicrobial resistance.

**B**acterial infections, such as bacteremia and pneumonia, have been associated with high rates of morbidity, mortality and economic costs, posing a real threat to public health globally. The mainstay of drug therapy for these infections is appropriate antibiotic treatment.^1^ Over the past 2 decades, however, the level of antimicrobial resistance of major bacterial species, such as *Escherichia coli* (*E. coli*) and *Pseudomonas aeruginosa [P. aeruginosa]*, has increased substantially.^2^ Moreover, multidrug resistance (MDR), defined as resistance to at least one agent in ≥3 antimicrobial groups, has increasingly been reported locally and globally. For instance, a recent study from Saudi Arabia has found that 67% of *E. coli* urine isolates were MDR.^3^ Multidrug resistance constitutes a stumbling block in the face of developing new therapeutics. Nevertheless, a combinatorial approach involving the use of established antimicrobial agents along with naturally-derived compounds has produced encouraging results.^4^

Quinones are aromatic dicarbonyl compounds that naturally exist in plants but can also be synthetically synthesized. Quinones have gained considerable attention for their medicinal properties including antimicrobial, anti-inflammatory, anti-atherosclerotic, and antitumor functions.^5^ Plumbagin, or 5-hydroxy-2-methyl-naphthalene-1,4-dione, is a yellow crystalline naphthoquinone derivative with a molar mass of 188.17 g/mol. A vitamin K3 analogue, PGN possesses an additional hydroxy group on carbon 5 in the naphthalene group (Figure 1). Plumbagin is present in the root, leaf, and stem bark of multiple plants including *Plumbaginaceae, Ebenceae, Dioncophyllaceae, Ancestrocladaceae, Droseraceae,* and *Juglandaceae families*.^6,7^ In particular, the roots of *Plumbago zeylanica L.* have been successfully used in India for therapeutic purposes against dermal and musculoskeletal conditions for more than 2500 years.^8^

**Figure 1:**
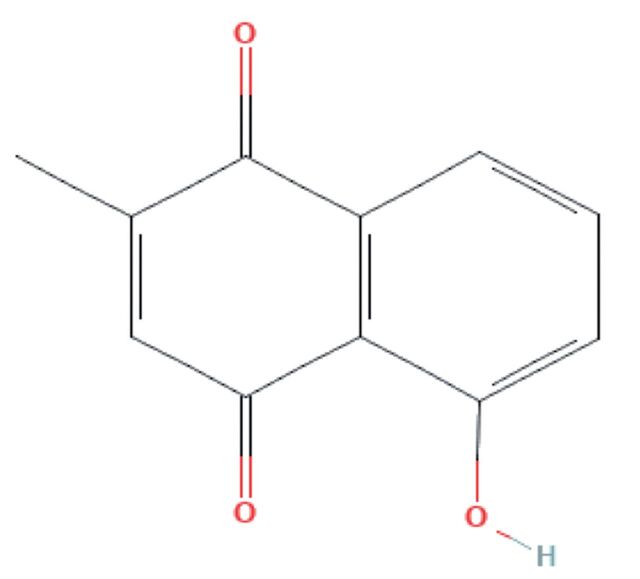
- Molecular structure of plumbagin.^39^ Characteristic hydroxyl and methyl groups added to para-naphthoquinone are noted.

Beside its anti-inflammatory, antioxidant, antitumor, and antidiabetic activities,previous reports have demonstrated the efficacy of PGN against pathogenic microbes.^9-12^ In particular, *Staphylococcus aureus [S. aureus]* and *Candida albicans* were susceptible to PGN *in vitro* and *in vivo*.^13,14^ Plumbagin also exhibited synergistic activity with ciprofloxacin and piperacillin against MDR strains of methicillin-resistant *S. aureus* (MRSA), which was through inhibition of DNA gyrase.^15,9^ Nevertheless, the potential efficacy of PGN against other bacterial species remains poorly studied.

Most studies were carried out using plant extracts with mixed chemical constituents, and little is known regarding PGN against MDR bacterial isolates in Saudi Arabia. In this report, we examine the antibacterial activity of PGN against clinical isolates of Gram-negative (*E. coli, Klebsiella pneumoniae [K. pneumoniae], Salmonella Typhi [S. Typhi],* and *P. aeruginosa*) and Gram-positive (*S. aureus*, *Staphylococcus saprophyticus [S. saprophyticus]*, *Streptococcus pyogenes [S. pyogenes*], and *Enterococcus faecalis [E. faecalis]*) bacteria in an attempt to further elucidate its potential application in antimicrobial therapy. We also sought to determine the anti-virulence potential of PGN through evaluating its anti-biofilm activity against these isolates.

## Methods

This is an experimental investigation that was carried out at the Department of Clinical Lab Sciences, College of Applied Medical Sciences, King Khalid University, Abha, Saudi Arabia, from October to December 2021. Ethical approval was obtained from the Research Ethics Committee at King Khalid University (ECM#2021-2801).

### Preparation of PGN stock solution

Plumbagin (CAS #481-42-5) from *P. zeylanica L* was obtained from Solarbio Life Science (Beijing, China) and the stock solution was prepared by suspending 1.88 µg in 1 mL of dimethyl sulfoxide (DMSO). Stock suspension was sonicated (Sonics Vibra cell; Sonics & Material, Newtown, CT, USA) at 40°C for 10 minutes and aliquots were preserved at –80°C. Working solutions were prepared by taking 1 mL of the stock solution and adding it to 9 mL of 2% DMSO. Further dilutions were made as necessary to achieve the required final concentration of PGN. Appendix 1 shows lack of antibacterial activity by DMSO on tested isolates.

### Bacterial strains and growth conditions

A panel of clinical MDR Gram-negative and Gram-positive bacterial strains, including *P. aeruginosa*, *E. coli*, *K. pneumoniae*, *S. Typhi*, *S. aureus*, *S. saprophyticus*, *S. pyogenes*, and *E. faecalis*, were used in this study (Table 1). These strains were grown in nutrient broth at 37°C for 24 h.

**Table 1:** - Characteristics of bacterial strains used in this study.

Bacterial strains	Sample source	Year of Isolation	Resistance
*Pseudomonas aeruginosa*	Wound	2019	Imipenem, Gentamicin, Ciprofloxacin
*Escherichia coli*	Urine		Amoxicillin/clavulanic acid, Ceftazidime, Ciprofloxacin
*Klebsiella pneumoniae*	Blood		Piperacillin/Tazobactam, Cefoxitin, Ciprofloxacin
*Salmonella Typhi*	Throat		Ciprofloxacin, Cefepime, Amoxicillin/clavulanic acid
*Staphylococcus aureus*	Nasal		Tetracycline, Ciprofloxacin, Trimethoprim-Sulfamethoxazol
*Staphylococcus saprophyticus*	Urethra		Nitrofurantoin, Penicillin, Tetracycline
*Streptococcus pyogenes*	Sputum		Tetracycline, Levofloxacin, Azithromycin
*Enterococcus faecalis*	Urethra		Penicillin, Tetracycline, Ciprofloxacin

### Antimicrobial susceptibility testing

A lawn culture of the bacterial inoculum (OD_610_ of 0.01) was grown on Mueller–Hinton agar (HiMedia Labs, Mumbai, India) with 14 disk antibiotics (Liofilchem, Livorno, Italy) in line with the guidelines of Clinical and Laboratory Standards Institute (CLSI) (16). Following incubation for 24 hours (h) at 37°C, the diameter of the clear zone of inhibition was subsequently measured in millimeters. *E. coli* ATCC 25922 and *S. aureus* ATCC 25923 were used as control strains.

### Antibacterial susceptibility assay by agar well diffusion

Bacterial strains were grown to the logarithmic phase (OD_610_ of 0.4-0.6) in nutrient broth and then diluted to a theoretical OD_610_ of 0.01. Agar well diffusion was used to probe the antibacterial efficacy of PGN.^17^ Briefly, wells of 6 mm diameter were formed in the nutrient agar using the cap of a sterile syringe and a lawn culture was formed on the agar from diluted cultures using a sterile cotton swab. Next, 20 µL of PGN (1.88 µg/mL) and DMSO were added to triplicate wells and the plates were incubated aerobically for 24 h at 37°C. Gentamicin and vancomycin were used as positive controls and distilled water as a negative control. The diameter of the clear zone of inhibition of bacterial growth including the well diameter was measured in millimeters.

### Determination of minimum inhibitory concentration (MIC) and minimum bactericidal concentration (MBC)

Minimum inhibitory concentration and MBC of PGN were determined as described by Wei et al^18^ with modification. Plumbagin concentrations were diluted 2-fold and ranged from 1.88 µg/mL to 0.014 µg/mL. To determine MIC, cultures were first grown to logarithmic phase and then further diluted in Mueller-Hinton broth to a theoretical OD_610_ of 0.01. Subsequently, 180 µL of all cultures were transferred to 96-well plates, 20 µL from the 2-fold dilution of PGN were loaded in triplicate wells, and 20 µL of DMSO served as control. Following aerobic incubation for 24 h at 37°C, each well was supplemented with 20 µL of alamar blue (Thermo Fisher, Watham, MA, USA) and observed at 1-hour intervals for the development of a pink color. The lowest concentration that did not cause a color change was recorded as the MIC. For the determination of MBC, 10 µL from wells with no color change were sub-cultured on nutrient agar and incubated aerobically for 24 h at 37°C. The minimum concentration of PGN at which no growth was observed was taken as the MBC for the examined strains.

### Growth inhibition

A colony count method was employed to determine the bactericidal effect of PGN. Approximately 10 µL of PGN were added to bacterial suspensions plated on nutrient agar using a spread plate method together with a negative control. The plates were observed for growth inhibition by counting colonies after incubation for 24 h at 37°C. The percentage of loss in viable cells was determined using the following equation:


$$\text{I}\left(\%\right)\frac{\left(\text{μC}-\text{μT}\right)}{\text{μC}}\times 100$$

Where I% = percentage of bacterial growth inhibition; µC = mean value of OD_610_ in control cells; and µT = mean value of OD_610_ in treated cells.

### Antibiofilm effect of PGN

Biofilm formation of the bacterial isolates was evaluated according Zhang et al^19^ with minor modifications. Briefly, 180 µL of fresh bacterial suspension (OD_610_=1.0) was added to 20 µL of different concentrations of PGN (MIC x0.5, MIC x1, MIC x2) in a 96-well plate, and the suspension was incubated for 24 h at 30°C without shaking to induce biofilm assembly. Untreated bacterial cells were used in each set of investigations as a negative control. After incubation, crystal violet was added and the absorbance was recorded at 488 nm.

### Time-kill kinetics assay

To investigate the effect of PGN on tested bacterial cells over time, 180 µL of the bacterial culture (OD_610_ of 0.01) was treated with 20 µL of different concentrations of PGN (MIC x0.5, MIC x1, MIC x2). A culture well with 20 µL of DMSO was considered as the control. The plates were further incubated aerobically at 37°C and OD was measured at 610 nm at 2-h intervals. The mean OD readings were plotted against time.

### Statistical analysis

Experiments were performed three times, and the results were plotted as mean ± SD. GraphPad Prism 6.0 (GraphPad Software, Inc., San Diego, CA, USA) was used for statistical analysis. Variations between 2 groups were examined with the 2-tailed Student’s t test, while one-way analysis of variance with Dunnett’s correction compared more than two groups. A *p*-value of <0.05 was considered statistically significant.

## Results

### Antibiotic susceptibility assay of standard antibiotics

To determine antibiotic susceptibility, clinical isolates of Gram-negative and Gram-positive bacteria including *P. aeruginosa, E. coli, K. pneumoniae, S. Typhi and S. aureus, S. saprophyticus, S. pyogenes,* and *E. faecalis* were tested against antibiotics shown in Tables 1 & 2. All of the tested bacterial strains were resistant to 3 or more of the standard antibiotics (Appendix 2 & 3) which indicates that they are MDR. Antibacterial activity of PGN

**Table 2:** - Details of antibiotics used in this study.

Antibiotic group	Antibiotic name	Concentration (µg/disc)
Penicillins	Penicillin	10
β-lactam/β-lactamase inhibitors combination	Amoxicillin-clavulanic acid	20/10
	Piperacillin/Tazobactam	100/10
Aminoglycosides	Gentamicin	10
Second generation cephalosporins	Cefoxitin	30
Third generation cephalosporins	Ceftazidime	30
Fourth generation cephalosporins	Cefepime	30
Tetracyclines	Tetracycline	30
Carbapenems	Imipenem	10
Fluoroquinolones	Ciprofloxacin	5
	Levofloxacin	5
Nitrofurans	Nitrofurantoin	300
Macrolides	Azithromycin	15
Folate pathway inhibitors	Trimethoprim-Sulfamethoxazol	1.25/23.75

**Figure 2:**
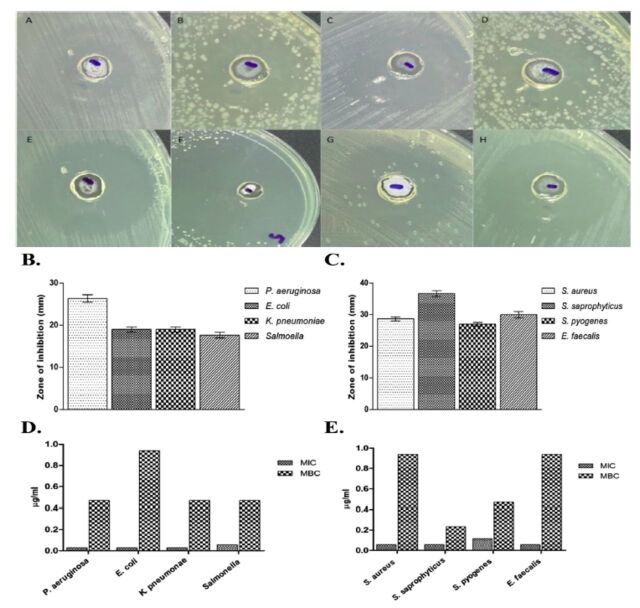
- Susceptibility of tested organisms to plumbagin (PGN). (**A-C**) Zone of inhibition is observed on plates supplemented with PGN against **A**) *Pseudomonas aeruginosa*, **B**) *Escherichia coli*, **C**) *Klebsiella pneumoniae*, **D**) *Salmonella*, **E**) *Staphylococcus aureus*, **F**) *Staphylococcus saprophyticus*, **G**) *Streptococcus pyogenes*, and **H**) *Enterococcus faecalis*. (**D & E**) MIC and MBC values of PGN against Gram-negative and Gram-positive isolates.

To assess the antibacterial activity of PGN, clinical isolates of Gram-negative and Gram-positive bacteria including *P. aeruginosa, E. coli, K. pneumoniae, S. Typhi and S. aureus, S. saprophyticus, S. pyogenes,* and *E. faecalis* were used. Susceptibility studies showed that PGN had a comparatively higher antibacterial activity against Gram-positive isolates. A zone size above 8 mm was considered significant based on the sensitivity of the bacterial strains to PGN. The zone of inhibition against Gram-positive bacteria was 28 to 35 mm in diameter, while that against Gram-negative bacteria ranged from 17 to 26 mm in diameter (Figure 2A-C).

In order to investigate MIC and MBC indices, selected bacterial strains were exposed to the aforementioned volume of PGN followed by an incubation period of 24 h. As shown in Figure 2, all bacterial strains tested were significantly susceptible to PGN. Bacterial growth was inhibited with higher zones of inhibition ranging from 17 to 35 mm, reflective of MIC values of 0.029-0.117 µg/mL and MBC values of 0.235-0.94 µg/mL (Figure 2D and 2E). For comparison, MIC and MBC values of gentamicin and vancomycin were used as controls against Gram-negative and Gram-positive isolates, respectively (Appendix 2).

### Plumbagin inhibits the viability of bacterial cells

**Figure 3:**
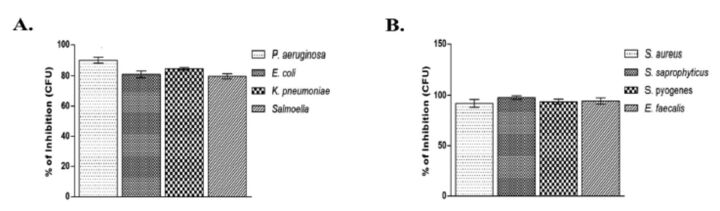
- Bacterial growth inhibition by plumbagin (PGN). Mean percentage of growth inhibition + SD by PGN at minimum bactericidal concentration for 24 h at 37°C for (**A**) Gram-negative and (**B**) Gram-positive isolates. Control bars indicate all untreated bacterial strains presented as 0% inhibition. CFU: colony-forming unit, *P. aeruginosa: Pseudomonas aeruginosa, E. coli: Escherichia coli, K. pneumoniae: Klebsiella pneumoniae*, *S*. *aureus*: *Staphylococcus aureus, S. saprophyticus: Staphylococcus saprophyticus, S. pyogenes: Streptococcus pyogenes, E. faecalis: Enterococcus faecalis*

Growth inhibitory effects of PGN on the tested bacterial strains were determined by counting the bacterial colonies after treating with PGN. The results of colony count on the tested bacterial strains showed a reduction in the number of colonies at the MBC concentration of PGN ranging from 79% to 99% (Figure 3). The lowest growth inhibition was found against *S. Typhi* (Figure 3A), whereas the highest was recorded against *S. saprophyticus* (Figure 3B).

### Plumbagin inhibits bacterial biofilm formation

We attempted to investigate whether PGN could inhibit biofilm formation of the tested Gram-negative and Gram-positive bacterial strains. Bacteria were incubated with MIC x0.5, MIC x1, and MIC x2 of PGN for 24 h at 37°C. Furthermore, the rate of biofilm formation inhibition by PGN was also based on the concentration and treatment time.

**Figure 4:**
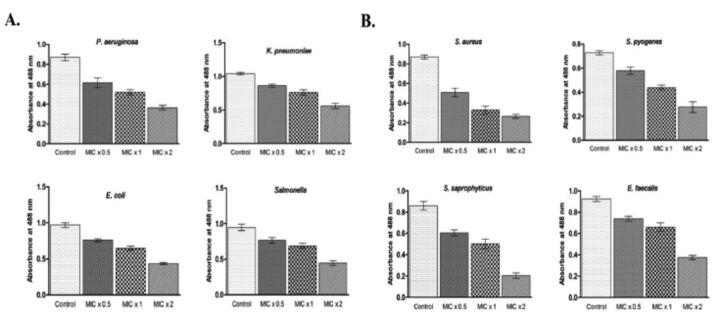
- Plumbagin (PGN) reduces biofilm formation. (**A**) Gram-negative and (**B**) Gram-positive isolates were treated with different concentrations of PGN (minimum inhibitory concentration [MIC] x0.5, MIC x1 and MIC x2) under biofilm growing conditions for 24 hours and mean absorbance readings at 488 nm + SD were plotted. *P. aeruginosa: Pseudomonas aeruginosa, E. coli: Escherichia coli, K. pneumoniae: Klebsiella pneumoniae*, *S*. *aureus*: *Staphylococcus aureus, S. saprophyticus: Staphylococcuss saprophyticus, S. pyogenes: Streptococcus pyogenes, E. faecalis: Enterococcus faecalis*

The inhibition of biofilm formation was significant against all the tested bacteria but it was drastically reduced against *S. saprophyticus* and *S. aureus* in the wells treated with MIC x2 of PGN. At MIC x2 of PGN, quantitative estimation of biofilms formed by *S. saprophyticus* showed a 4.2-fold decrease and by *S. aureus* a 3.01-fold decrease, compared to control values (Figure 4 A&B). These results suggest that biofilm formation by all the tested bacteria was inhibited by PGN.

### Effect on bacterial growth

**Figure 5:**
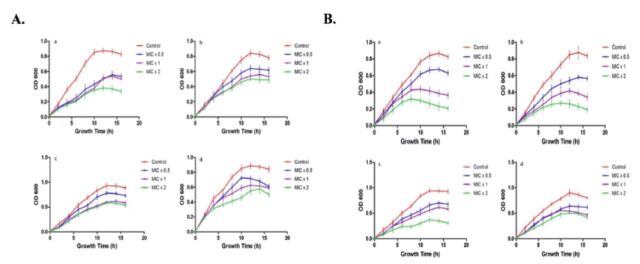
- Kinetics of plumbagin (PGN)-induced growth inhibition. Representative (**A**) Gram-negative (**a**. *Pseudomonas aeruginosa*, **b**. *Escherichia coli*, **c**. *Klebsiella pneumoniae*, **d**. Salmonella) and (**B**) Gram-positive (**a**. *Staphylococcus aureus*, **b**. *Staphylococcus saprophyticus*, **c**. *Streptococcus pyogenes*, **d**. *Enterococcus faecalis*) strains were treated with different concentrations (MIC x0.5, MIC x1 and MIC x2) of PNG. Growth cycle of untreated organisms served as control. OD_610_ nm was measured at regular intervals of 2 hours and presented as mean + SD.

To determine time-killing kinetics of PGN, 180 µL of bacterial culture (OD_610_ of 0.01) were treated with 20 µL of PGN at different concentrations of MIC x0.5, MIC x1, and MIC x2. The growth of bacteria was observed at time intervals of 2 h (Figure 5). It is absolutely apparent that bacterial strain growth was repressed by treatment with PGN at different concentrations. Time killing kinetics indicated a dose dependent bactericidal effect of PGN on the tested bacteria. Our data distinctly indicates a strong bactericidal activity of PGN against the tested bacterial strains.

## Discussion

Despite making great strides in the combat against microbial resistance, MDR remains a pivotal challenge in the development and validation of novel antimicrobial therapeutics. This is mainly due to the widespread inappropriate use of current antibiotics, in addition to the stagnated discovery of new efficacious chemotherapeutic agents.^9^ Although the antimicrobial activity of *P. zeylanica* extracts is well established in the literature, the antibacterial activity of PGN, a major constituent of this plant, has not been thoroughly investigated. Herein, we demonstrate that PGN exhibits a potent, broad-spectrum antibacterial activity against clinical isolates of major worldwide concern.

The zones of inhibition against Gram-positive bacteria were larger than those against Gram-negative isolates (Figure 2), which could be attributed to the presence of a physical cell wall barrier in the latter as well as the presence of to efflux pumps. This is in parallel to the results obtained by Dissanayake et al^9^ using *P. indica* extract. It is worth mentioning that standard antibiotics exhibit MIC values ranging from 15-107 µg/mL20 which, compared to our findings of 0.029-0.117 µg/mL (Figure 2), highlights the potent antibacterial activity of PGN. We have previously shown that thymoquinone had an MIC of 1.04-8.3 µg/mL, reflective of a much weaker activity.^4^ Furthermore, our data indicate that PGN compares favorably and exceeds in antimicrobial potency a panel of alkaloid, terpenoid, phenolic, and thiophene natural products.^21^

The highest susceptibility among the tested strains was observed against *S. saprophyticus* (Figure 2D). This coagulase-negative pathogen is responsible for urinary tract infections (UTIs) and related genitourinary complications in susceptible hosts with preexisting conditions.^22^ Behind only *E. coli, S. saprophyticus* is the second most frequently encountered cause of uncomplicated community-acquired UTIs in females.^23^ Currently, nitrofurantoin and a combination of trimethoprim-sulfamethoxazole are the antimicrobials of choice for *S. saprophyticus* infections. However, major side effects of these drugs include fatal pulmonary, dermal, gastrointestinal, hematological, and neurological symptoms.^24^ Thus, in light of available evidence, PGN may offer a safer alternative for *S. saprophyticus* infections.

Conversely, although shown to be susceptible, *E. coli, S. aureus*, and *E. faecalis* required the highest concentrations of PGN compared to the other tested strains (Figure 2 D&E). This is in agreement with the consensus on these organisms being notorious for rapid acquisition of resistance.^4^ Pathogenic E. coli, including intestinal and extraintenstinal strains, are behind the etiology of diarrhea, UTIs, septicemia, and meningitis, and adhesins, release of toxins, and tissue invasion form the major virulence factors mediating pathogenesis. MDR *E. coli* strains are responsible for both nosocomial and community-acquired infections, and they are often resistant to front-line agents, such as penicillin and third-generation cephalosporins. This resistance, mediated by extended-spectrum β-lactamase (ESBL),^25^ has now increased due to the worldwide dissemination of the specific *E. coli* clone, *E. coli* ST131.^26^ In an attempt to tackle this resistance issue, more powerful agents, such as carbapenems and polymyxins, have recently been relaunched as alternatives to inactive antibiotics. However, carbapenems suffer from poor absorbability, and cause pruritis, hepatotoxicity, diarrhea, vomiting, and nausea.^27^

Virulent strains of *S. aureus*s cause a variety of fatal pathologies including osteoarthritis, endocarditis, sepsis, and prosthesis-related infections, among others.^28^ Compared to glycopeptides such as vancomycin, methicillin-sensitive *S. aureus* responds well to β-lactam antibiotics, but this is not without relapse.^29,30^ For MRSA, vancomycin and daptomycin remain the gold standard therapy. Nevertheless, the continuing emergence of resistant strains, combined with the established toxicity of these drugs and their prolonged use,underpins the search for alternative, fast-acting, and less harmful alternatives. Importantly, septic arthritis is often managed with immunosuppressants such as dexamethasone, which increases the patient’s susceptibility to infections and predisposes to hyperglycemia, an effect counteracted by PGN.^10,31,32^

Clinical isolates of *E. faecalis* cause a host of ailments ranging from UTIs and bacteremia to endocarditis and meningitis. A recognized threat to public health, *E. faecalis* is most commonly resistant to rifampicin and erythromycin, in addition to vancomycin, which surprisingly may serve as a growth factor.^33,34^ *Enterococcus faecalis* is also able to reside in biofilms,^35^ further aggravating its virulence. Our results indicate that PGN possesses antibiofilm activity (Figure 4). Biofilm formation aids in immune evasion, which fosters prolonged colonization and tissue injury. Moreover, cells embedded in biofilms often remain in a dormant state, having their metabolism operating at a lower rate. As a consequence, biofilm-associated infections often respond variably to available therapy. These infections affect virtually all body systems, and, alarmingly, may promote tumorigenesis.^36^ Therefore, modulating biofilm assembly and persistence, possibly through adhesins and quorum sensing, may prove to be efficacious against a multitude of life-threatening ailments. Of note, a number of polyphenols, including coumarins, anthocyanins, tannins, and flavonoids have also been demonstrated to interfere with biofilm formation in *S. aureus, K. pneumoniae, P. aeruginosa, E. coli,* and *E. faecalis*.^37^

Development of pharmaceutical formulations relies on interaction of the active ingredient with excipients that could either enhance or suppress its efficacy. Notably, the bark of *P. zeylanica* has successfully been used to synthesize silver and gold nanoparticles with discernable antibacterial activity against *P. aeruginosa* and *B. subtilis*.^38^ Therefore, evaluating the antimicrobial activity of PGN in nanoparticle formulations, and in the presence of common excipients such as polyethylene glycol, is also warranted.

### Study limitations

The current study lacks *in vivo* confirmation, effects of long-term exposure, and biochemical and molecular mechanisms, in addition to unknown additive or antagonistic interactions of PGN with other antibacterial compounds. Future studies should investigate the possible additive or synergistic role of PGN with standard antibiotics as well as the range of susceptible organisms and the mechanisms involved in its antimicrobial and anti-virulence activities both *in vitro* and *in vivo*.

In conclusion, since the majority of previous studies focused on plant extracts with mixed constituents, our report identifies PGN as a stand-alone broad-spectrum antibacterial agent effective against major clinical bacterial isolates from Saudi Arabia. Plumbagin is demonstrated to possess superior potency over numerous established and investigative antimicrobials, in addition to antibiofilm activity.

## Figures and Tables

**Appendix 1:**
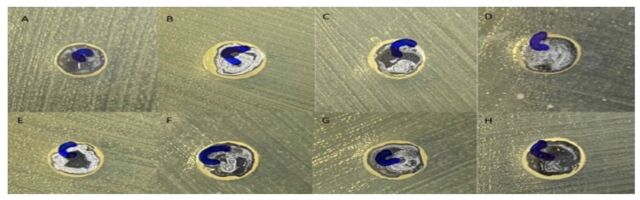
- Lack of effect of dimethyl sulfoxide on bacterial growth. Zone of inhibition is observed on plates for **A**) *Pseudomonas aeruginosa*, **B**)*Escherichia coli*, **C**) *Klebsiella pneumoniae*, **D**) *Salmonella Typhi*, **E**) *Staphylococcus aureus*, **F**) *Staphylococcus saprophyticus*, **G**) *Streptococcus pyogenes*, and **H**) *Enterococcus faecalis.*

**Appendix 4:**
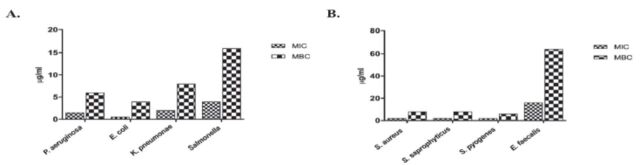
- Minimum inhibitory concentration (MIC) and minimum bacterial concentration (MBC) values of gentamicin and vancomycin. Gram-negative **A**) and Gram-positive **B**) isolates were treated with gentamicin and vancomycin, respectively, to determine MIC and MBC values. *P. aeruginosa: Pseudomonas aeruginosa, E. coli: Escherichia coli, K. pneumoniae: Klebsiella pneumoniae*, *S*. *Typhi: Salmonella typhi, S*. *aureus*: *Staphylococcus aureus, S. saprophyticus: Staphylococcus saprophyticus, S. pyogenes: Streptococcus pyogenes, E. faecalis: Enterococcus faecalis,*

## Appendix

**Appendix 2:** - Resistance patterns of Gram-negative bacterial strains used in the study.

Bacterial strain	Zone of inhibition (mm)							
	AUG (20/10 µg)	Piperacillin/Tazobactam (100/10 µg)	Cefoxitin (30 µg)	Ceftazidime (30 µg)	Cefepime (30 µg)	Imipenem (10 µg)	Gentamicin (10 µg)	Ciprofloxacin (5 µg)
*P. aeruginosa*	R	R	15	15	15	R	R	R
*E. coli*	R	R	R	R	R	25	22	R
*K. pneumoniae*	R	R	R	R	R	24	18	R
*S. typhi*	R	R	R	R	R	20	R	R

*P. aeruginosa: Pseudomonas aeruginosa, E. coli: Escherichia coli, K. pneumoniae: Klebsiella pneumoniae, S. Typhi: Salmonella typhi,* AUG: Amoxicillin-clavulanic acid, R: resistant

**Appendix 3:** - Resistance patterns of Gram-positive bacterial strains used in the study.

Bacterial strain	Zone of inhibition (mm)						
	SXT (1.25/23.75µg)	Tetracycline (30 µg)	Ciprofloxacin (5 µg)	Penicillin (10 µg)	Nitrofurantoin (300 µg)	Levofloxacin (5µg)	Azithromycin (15 µg)
S. aureus	R	R	R	R	18	R	18
S. saprophyticus	24	R	22	R	R	21	22
S. pyogenes	25	R	R	R	20	R	R
E. faecalis	R	R	R	R	25	R	23

*S. aureus: Staphylococcus aureus, S. saprophyticus: Staphylococcus saprophyticus, S. pyogenes: Streptococcus pyogenes, E. faecalis: Enterococcus faecalis,* R: resistant

## References

[1] File TM, Jr., Niederman MS. Antimicrobial therapy of community-acquired pneumonia. Infect Dis Clin North Am 2004; 18: 993–1016, xi. 15555836 10.1016/j.idc.2004.07.011 PMC7118969

[2] Croxall G , Weston V , Joseph S , Manning G , Cheetham P , McNally A. Increased human pathogenic potential of Escherichia coli from polymicrobial urinary tract infections in comparison to isolates from monomicrobial culture samples. J Med Microbiol 2011; 60: 102–109. 20947667 10.1099/jmm.0.020602-0

[3] Alqasim A , Abu Jaffal A , Alyousef AA. Prevalence of multidrug resistance and extended-spectrum beta-lactamase carriage of clinical uropathogenic *Escherichia coli* isolates in Riyadh, Saudi Arabia. Int J Microbiol 2018; 2018: 3026851. 30305814 10.1155/2018/3026851 PMC6165594

[4] Dera AA , Ahmad I , Rajagopalan P , Shahrani MA , Saif A , Alshahrani MY , et al. Synergistic efficacies of thymoquinone and standard antibiotics against multi-drug resistant isolates. Saudi Med J 2021; 42: 196–204. 33563739 10.15537/smj.2021.2.25706 PMC7989283

[5] Xu K , Wang P , Wang L , Liu C , Xu S , Cheng Y , et al. Quinone derivatives from the genus Rubia and their bioactivities. Chem Biodivers 2014; 11: 341–363. 24634067 10.1002/cbdv.201200173

[6] Shukla S , Wu CP , Nandigama K , Ambudkar SV. The naphthoquinones, vitamin K3 and its structural analogue plumbagin, are substrates of the multidrug resistance linked ATP binding cassette drug transporter ABCG2. Mol Cancer Ther 2007; 6: 3279–3286. 18065489 10.1158/1535-7163.MCT-07-0564 PMC2398729

[7] Liu Y , Cai Y , He C , Chen M , Li H. Anticancer properties and pharmaceutical applications of plumbagin: A review. Am J Chin Med 2017; 45: 423–441. 28359198 10.1142/S0192415X17500264

[8] Hafeez BB , Zhong W , Mustafa A , Fischer JW , Witkowsky O , Verma AK. Plumbagin inhibits prostate cancer development in TRAMP mice via targeting PKCepsilon, Stat3 and neuroendocrine markers. Carcinogenesis 2012; 33: 2586–2592. 22976928 10.1093/carcin/bgs291 PMC3510739

[9] Dissanayake D , Perera D , Keerthirathna LR , Heendeniya S , Anderson RJ , Williams DE , et al. Antimicrobial activity of Plumbago indica and ligand screening of plumbagin against methicillin-resistant *Staphylococcus aureus* . J Biomol Struct Dyn 2020; 40: 3273–3284. 33213303 10.1080/07391102.2020.1846622

[10] Sunil C , Duraipandiyan V , Agastian P , Ignacimuthu S. Antidiabetic effect of plumbagin isolated from Plumbago zeylanica L. root and its effect on GLUT4 translocation in streptozotocin-induced diabetic rats. Food Chem Toxicol 2012; 50: 4356–4363. 22960630 10.1016/j.fct.2012.08.046

[11] Likhitwitayawuid K , Kaewamatawong R , Ruangrungsi N , Krungkrai J. Antimalarial naphthoquinones from Nepenthes thorelii. Planta Med 1998; 64: 237–241. 9581522 10.1055/s-2006-957417

[12] Fournet A , Angelo A , Munoz V , Roblot F , Hocquemiller R , Cave A. Biological and chemical studies of Pera benensis, a Bolivian plant used in folk medicine as a treatment of cutaneous leishmaniasis. J Ethnopharmacol 1992; 37: 159–164. 1434690 10.1016/0378-8741(92)90074-2

[13] de Paiva SR , Figueiredo MR , Aragao TV , Kaplan MA. Antimicrobial activity in vitro of plumbagin isolated from Plumbago species. Mem Inst Oswaldo Cruz. 2003; 98: 959–961. 14762525 10.1590/s0074-02762003000700017

[14] Nair SV , Baranwal G , Chatterjee M , Sachu A , Vasudevan AK , Bose C , et al. Antimicrobial activity of plumbagin, a naturally occurring naphthoquinone from Plumbago rosea, against Staphylococcus aureus and Candida albicans. Int J Med Microbiol 2016; 306: 237–248. 27212459 10.1016/j.ijmm.2016.05.004

[15] Periasamy H , Iswarya S , Pavithra N , Senthilnathan S , Gnanamani A. In vitro antibacterial activity of plumbagin isolated from Plumbago zeylanica L. against methicillin-resistant Staphylococcus aureus. Lett Appl Microbiol 2019; 69: 41–49. 31044446 10.1111/lam.13160

[16] Clinical Laboratory Standards Institute. Performance Standards for Antimicrobial Susceptibility Testing. CLSI supplement M100. 28th ed. Wayne, PA, USA: Clinical and Laboratory Standards Institute; 2018.

[17] Magaldi S , Mata-Essayag S , Hartung de Capriles C , Perez C , Colella MT , Olaizola C , et al. Well diffusion for antifungal susceptibility testing. Int J Infect Dis 2004; 8: 39–45. 14690779 10.1016/j.ijid.2003.03.002

[18] Wei JR , Krishnamoorthy V , Murphy K , Kim JH , Schnappinger D , Alber T , et al. Depletion of antibiotic targets has widely varying effects on growth. Proc Natl Acad Sci U S A 2011; 108: 4176–4181. 21368134 10.1073/pnas.1018301108 PMC3053961

[19] Zhang L , Xu J , Xu J , Zhang H , He L , Feng J. TssB is essential for virulence and required for type VI secretion system in Ralstonia solanacearum. Microb Pathog 2014; 74: 1–7. 24972114 10.1016/j.micpath.2014.06.006

[20] Cermak P , Olsovska J , Mikyska A , Dusek M , Kadleckova Z , Vanicek J , et al. Strong antimicrobial activity of xanthohumol and other derivatives from hops (Humulus lupulus L.) on gut anaerobic bacteria. APMIS 2017; 125: 1033–1038. 28960474 10.1111/apm.12747

[21] Mbaveng AT , Sandjo LP , Tankeo SB , Ndifor AR , Pantaleon A , Nagdjui BT , et al. Antibacterial activity of nineteen selected natural products against multi-drug resistant Gram-negative phenotypes. Springerplus 2015; 4: 823. 26753111 10.1186/s40064-015-1645-8 PMC4695461

[22] Pinault L , Chabriere E , Raoult D , Fenollar F. Direct identification of pathogens in urine by use of a specific matrix-assisted laser desorption ionization-time of flight spectrum database. J Clin Microbiol 2019; 57. 10.1128/JCM.01678-18 PMC6440795 30700506

[23] Hur J , Lee A , Hong J , Jo WY , Cho OH , Kim S , et al. *Staphylococcus saprophyticus* bacteremia originating from urinary tract infections: A case report and literature review. Infect Chemother 2016; 48: 136–139. 27433385 10.3947/ic.2016.48.2.136 PMC4945724

[24] Karpman E , Kurzrock EA. Adverse reactions of nitrofurantoin, trimethoprim and sulfamethoxazole in children. J Urol 2004; 172: 448–453. 15247700 10.1097/01.ju.0000130653.74548.d6

[25] Makvana S , Krilov LR. Escherichia coli Infections. Pediatr Rev 2015; 36: 167–170; quiz 71. 25834220 10.1542/pir.36-4-167

[26] Alqasim A. Colistin-resistant Gram-genative bacteria in Saudi Arabia: A literature review. J King Saud Univ Sci 2021; 33: 101610.

[27] Carbapenems. LiverTox: Clinical and Research Information on Drug-Induced Liver Injury. Bethesda (MD) 2012. 31643176

[28] Tong SY , Davis JS , Eichenberger E , Holland TL , Fowler VG , Jr. Staphylococcus aureus infections: epidemiology, pathophysiology, clinical manifestations, and management. Clin Microbiol Rev 2015; 28: 603–661. 26016486 10.1128/CMR.00134-14 PMC4451395

[29] Pragman AA , Kuskowski MA , Abraham JM , Filice GA. Infectious disease consultation for *Staphylococcus aureus* bacteremia improves patient management and outcomes. Infect Dis Clin Pract (Baltim Md) 2012; 20: 261–267. 23049234 10.1097/IPC.0b013e318255d67c PMC3464014

[30] Chong YP , Moon SM , Bang KM , Park HJ , Park SY , Kim MN , et al. Treatment duration for uncomplicated Staphylococcus aureus bacteremia to prevent relapse: analysis of a prospective observational cohort study. Antimicrob Agents Chemother 2013; 57: 1150–1156. 23254436 10.1128/AAC.01021-12 PMC3591920

[31] Moise PA , North D , Steenbergen JN , Sakoulas G. Susceptibility relationship between vancomycin and daptomycin in *Staphylococcus aureus*: facts and assumptions. Lancet Infect Dis 2009; 9: 617–624. 19778764 10.1016/S1473-3099(09)70200-2

[32] Arti H , Mousapour A , Alavi SM. The effect of intravenous dexamethasone in the treatment of septic arthritis. Pak J Med Sci 2014; 30: 955–957. 25225506 10.12669/pjms.305.5217 PMC4163211

[33] Jahansepas A , Aghazadeh M , Rezaee MA , Hasani A , Sharifi Y , Aghazadeh T , et al. Occurrence of *Enterococcus faecalis and Enterococcus faecium* in various clinical infections: detection of their drug resistance and virulence determinants. Microb Drug Resist 2018; 24: 76–82. 28525287 10.1089/mdr.2017.0049

[34] Sukumaran V , Cosh J , Thammavong A , Kennedy K , Ong CW. Vancomycin dependent *Enterococcus*: an unusual mutant? Pathology 2019; 51: 318–320. 30846227 10.1016/j.pathol.2018.11.012

[35] Ch’ng JH , Chong KKL , Lam LN , Wong JJ , Kline KA. Biofilm-associated infection by enterococci. Nat Rev Microbiol 2019; 17: 82–94. 30337708 10.1038/s41579-018-0107-z PMC13729018

[36] Li S , Konstantinov SR , Smits R , Peppelenbosch MP. Bacterial biofilms in colorectal cancer initiation and progression. Trends Mol Med 2017; 23: 18–30. 27986421 10.1016/j.molmed.2016.11.004

[37] Slobodnikova L , Fialova S , Rendekova K , Kovac J , Mucaji P. Antibiofilm activity of plant polyphenols. Molecules 2016; 21: 1717. 27983597 10.3390/molecules21121717 PMC6273306

[38] Velammal SP , Devi TA , Amaladhas TP. Antioxidant , antimicrobial and cytotoxic activities of silver and gold nanoparticles synthesized using Plumbago zeylanica bark. J Nanostructure Chem 2016; 6: 247–260.

[39] PubChem. Bethesda (MD): National Library of Medicine (US), National Center for Biotechnology Information; PubChem Compound Summary for CID 10205, Plumbagin. [Updated 2004; cited 2022 Oct 25]. Available from: https://pubchem.ncbi.nlm.nih.gov/compound/Plumbagin

